# Atg44/Mdi1/mitofissin facilitates Dnm1-mediated mitochondrial fission

**DOI:** 10.1080/15548627.2024.2360345

**Published:** 2024-05-31

**Authors:** Kentaro Furukawa, Manabu Hayatsu, Kentaro Okuyama, Tomoyuki Fukuda, Shun-Ichi Yamashita, Keiichi Inoue, Shinsuke Shibata, Tomotake Kanki

**Affiliations:** a Department of Cellular Physiology, Graduate School of Medical Sciences, Kyushu University, Fukuoka, Japan; b Department of Cellular Physiology, Niigata University Graduate School of Medical and Dental Sciences, Niigata, Japan; c Division of Microscopic Anatomy, Niigata University Graduate School of Medical and Dental Sciences, Niigata, Japan

**Keywords:** Atg44, Dnm1, mitochondrial fission, mitofissin, mitophagy, yeast

## Abstract

Mitochondria undergo fission and fusion, and their coordinated balance is crucial for maintaining mitochondrial homeostasis. In yeast, the dynamin-related protein Dnm1 is a mitochondrial fission factor acting from outside the mitochondria. We recently reported the mitochondrial intermembrane space protein Atg44/mitofissin/Mdi1/Mco8 as a novel fission factor, but the relationship between Atg44 and Dnm1 remains elusive. Here, we show that Atg44 is required to complete Dnm1-mediated mitochondrial fission under homeostatic conditions. Atg44-deficient cells often exhibit enlarged mitochondria with accumulated Dnm1 and rosary-like mitochondria with Dnm1 foci at constriction sites. These mitochondrial constriction sites retain the continuity of both the outer and inner membranes within an extremely confined space, indicating that Dnm1 is unable to complete mitochondrial fission without Atg44. Moreover, accumulated Atg44 proteins are observed at mitochondrial constriction sites. These findings suggest that Atg44 and Dnm1 cooperatively execute mitochondrial fission from inside and outside the mitochondria, respectively.

**Abbreviation:** ATG: autophagy related; CLEM: correlative light and electron microscopy; EM: electron microscopy; ER: endoplasmic reticulum; ERMES: endoplasmic reticulum-mitochondria encounter structure; GA: glutaraldehyde; GFP: green fluorescent protein; GTP: guanosine triphosphate: IMM: inner mitochondrial membrane; IMS: intermembrane space; OMM: outer mitochondrial membrane; PB: phosphate buffer; PBS: phosphate-buffered saline; PFA: paraformaldehyde; RFP: red fluorescent protein; WT: wild type.

## Introduction

Mitochondria, the powerhouse of the cell, are highly dynamic organelles that undergo fission and fusion, and these mitochondrial dynamics are crucial for maintaining mitochondrial homeostasis [1]. Defects in the regulation of mitochondrial dynamics are associated with various human diseases [2], and extensive studies have been conducted to understand identified the regulatory factors and mechanisms underlying fission and fusion. Mitochondrial fission and fusion are regulated by distinct GTPases; fission is mediated by Dnm1 [3,4] in yeast and DNM1L/Drp1 [5] in mammals, while fusion is mediated by Fzo1 [6] and Mgm1 [7] in yeast and MFN1-MFN2 [8] and OPA1 [9] in mammals.

Before mitochondrial fission in yeast, contact sites form between the endoplasmic reticulum (ER) and mitochondria, and ER-mediated mitochondrial pre-constriction occurs [10,11]. Then, Dnm1 is recruited to the outer mitochondrial membrane (OMM) via binding to the receptor protein Fis1 [12]. Dnm1 forms an oligomeric ring-shaped structure and further constricts the mitochondria in a guanosine triphosphate (GTP) hydrolysis-dependent manner [13], eventually resulting in mitochondrial fission. However, it remains controversial whether mitochondrial constriction by Dnm1 and DNM1L is sufficient to complete fission. Lee *et al*. reported that, in mammalian cells, completion of mitochondrial fission requires both DNM1L and DNM2/DYN2 (dynamin 2) [14], while others reported that DNM2 is dispensable for mitochondrial fission [15,16]. Moreover, extensive *in vitro* experiments support different conclusions regarding the completion of membrane fission by Dnm1 and DNM1L [15,17–20]. Thus, it has remained unclear whether the mitochondrial constriction by Dnm1 and DNM1L leads to the complete severance of mitochondrial membranes or whether additional fission factors/mechanisms exist.

We recently reported that the intermembrane space (IMS) protein Atg44/mitofissin (also known as Mdi1 [21]/Mco8 [22]) drives mitochondrial fission during mitochondrial autophagy (mitophagy) in yeast and that Atg44 directly binds to lipid membranes and brings about membrane fragility to facilitate membrane fission [23]. Both Atg44-deficient and Dnm1-deficient yeast cells are defective in mitochondrial fission under homeostatic conditions, but the relationship between these two differently localized fission factors has remained unclear [24]. In this study, we demonstrate that Atg44 is required for the completion of Dnm1-mediated mitochondrial fission under homeostatic conditions. During the final stage of preparation of this manuscript, Connor et al. reported the identification of a protein, Mitochondrial Division IMS 1 (Mdi1; same as Atg44), required for Dnm1-mediated mitochondrial fission [21]. Our and their findings shed light on the coordinated function of these two fission factors operating both from outside and inside the mitochondria.

## Results

### Atg44 is required to complete Dnm1-mediated mitochondrial fission

**Figure 1.**
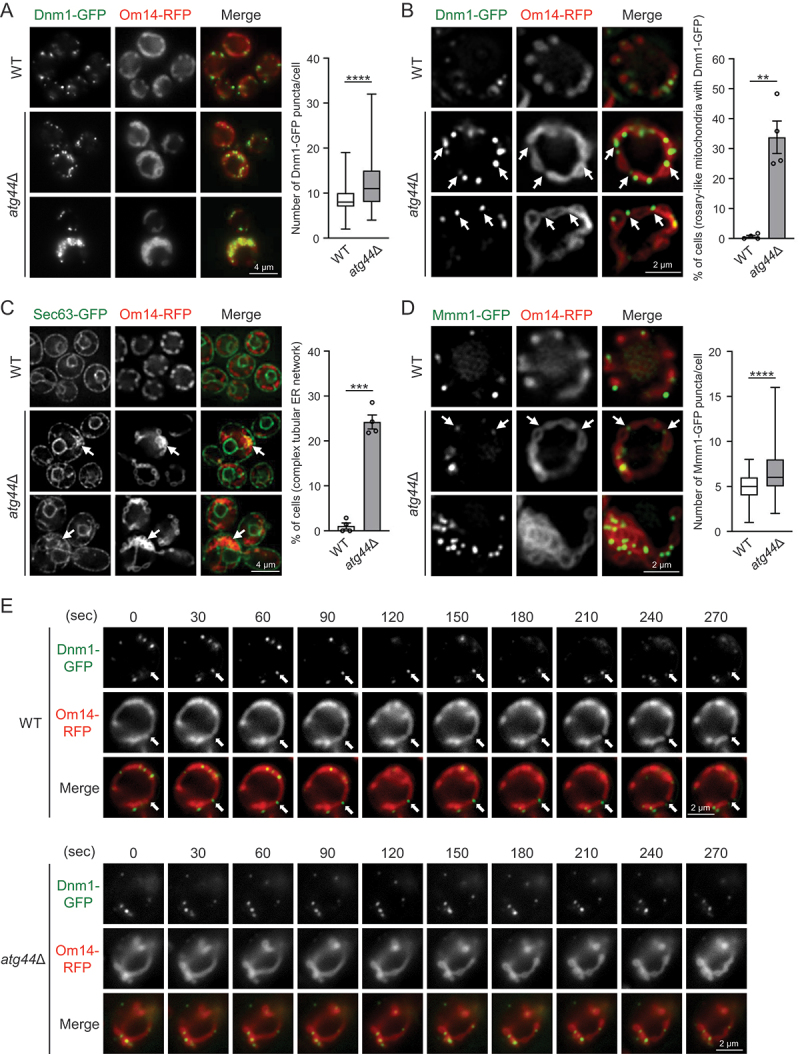
Atg44 is required to complete Dnm1-mediated mitochondrial fission. (A–D) S. cerevisiae WT and atg44∆ cells expressing Om14-RFP and the indicated GFP-fused proteins were cultured in YPL until mid-log phase and analyzed by fluorescence microscopy. (A) Typical mitochondrial morphology and distribution of Dnm1-GFP puncta in WT and atg44∆ cells. Enlarged or aggregated mitochondria with numerous Dnm1-GFP puncta in atg44∆ cells are shown in the lower panel. The number of Dnm1-GFP puncta associated with mitochondria was counted and the results are shown as boxplots. The boxplots indicate minimum, lower quartile, median, upper quartile, and maximum. ****p < 0.0001 (Welch’s t-test). WT, n = 480; atg44∆, n = 443; four experiments. (B) Fragmented mitochondria in WT cells and rosary-like mitochondria in atg44∆ cells. Bars show the percentage of cells with rosary-like mitochondria (no fragmented mitochondria) containing Dnm1-GFP puncta (white arrows). **p < 0.01 (Welch’s t-test). WT, n = 480; atg44∆, n = 510; four experiments. (C) Association of the complex tubular ER network with aggregated mitochondria in atg44∆ cells. Bars show the percentage of cells with complex tubular ER network on aggregated mitochondria (white arrows). ***p < 0.001 (Welch’s t-test). WT, n = 272; atg44∆, n = 249; four experiments. (D) Association of ERMES visualized by Mmm1-GFP with the mitochondrial constriction sites (white arrows, middle panel) or aggregated mitochondria (lower panel) in atg44∆ cells. The number of Mmm1-GFP puncta was counted and the results are shown as in (A). ****p < 0.0001 (Welch’s t-test). WT, n = 360; atg44∆, n = 360; three experiments. (E) Time-lapse images of Dnm1-mediated mitochondrial fission were acquired at 30-s intervals for a total of 270 s, 4 ± 1 h after shifting cells from YPD to YPL medium. A total of 2931 WT cells and 2998 atg44∆ cells were analyzed. White arrows indicate the fission site linked to Dnm1-GFP puncta. Scale bars are shown in each panel. See also Figures S1 and S2.

*S. cerevisiae* wild-type (WT) cells typically exhibit tubular mitochondria during fermentation (glucose medium) and fragmented mitochondria under respiratory growth conditions (lactate medium), while both Atg44-deficient (*atg44*∆) and Dnm1-deficient (*dnm1*∆) cells exhibit enlarged or swollen mitochondria under both conditions (Figure S1) [23]. These results indicate that mitochondrial fragmentation requires both Atg44 and Dnm1. In addition, the similarity of mitochondrial morphology in *atg44*∆ and *dnm1*∆ cells suggests a functional link between Atg44 and Dnm1. To investigate their relationship, we first examined whether the localization of Dnm1 is affected by the absence of Atg44. To this end, we co-expressed a C-terminal fusion of green fluorescent protein (GFP) with Dnm1 (Dnm1-GFP) and a C-terminal fusion of red fluorescent protein (RFP) with the OMM marker protein Om14 (Om14-RFP) in WT and *atg44*∆ cells. In both cell types, Dnm1-GFP puncta formed and nearly all were associated with mitochondria, regardless of their shape and size (Figure 1A), indicating that Atg44 is dispensable for the recruitment of Dnm1 to mitochondria. Dnm1-GFP puncta were more abundant in *atg44*∆ cells than in WT cells, and this increase is consistent with the observation that *atg44*∆ cells often exhibit enlarged or aggregated mitochondria associated with numerous puncta on their surface (Figure 1A, lower panel).

We found another difference in the mitochondrial morphology and distribution of Dnm1-GFP between WT and *atg44*∆ cells. In contrast to the fragmented mitochondria of WT cells, the mitochondria of *atg44*∆ cells often exhibited a rosary (prayer beads)-like morphology with a single Dnm1-GFP punctum at the constriction sites between mitochondrial masses (Figure 1B). This morphology appears to reflect an arrested state just before the completion of mitochondrial fission. Moreover, ER tubules visualized by Sec63-GFP (Figure 1C) and ER-mitochondria encounter structure (ERMES) visualized by Mmm1-GFP (Figure 1D) were also observed in abundance at aggregated mitochondria or mitochondrial constriction sites in *atg44∆* cells compared with WT cells. These results suggest that the ER continues to associate with mitochondria in *atg44∆* cells. Notably, the absence of Atg44 did not affect Dnm1 and Mmm1 protein levels (Figures S1C and S1D). Next, we performed time-lapse imaging to demonstrate that Atg44 is required for Dnm1-mediated mitochondrial fission. As shown in Figures 1E and S2, mitochondrial fission events were observed at the Dnm1-GFP puncta in WT cells (46 events in 2931 cells during each 270-s observation), whereas no such events were observed in *atg44∆* cells (in 2998 cells during each 270-s observation). Taken together, these results suggest that, despite the substantial focal recruitment of Dnm1 to mitochondria, Dnm1 is unable to complete mitochondrial fission without Atg44.

### CLEM imaging of the mitochondrial constriction sites with Dnm1-GFP signals

**Figure 2.**
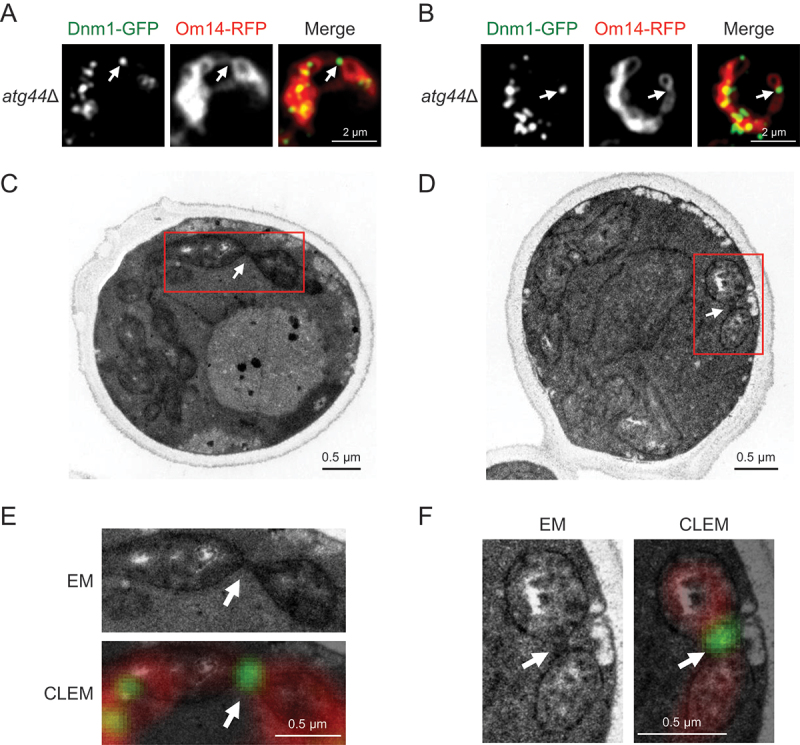
CLEM imaging of the mitochondrial constriction sites with Dnm1-GFP signals. S. cerevisiae atg44∆ cells expressing Dnm1-GFP and Om14-RFP were cultured in YPL until mid-log phase, fixed with 2% PFA/0.1% GA for 30 min at room temperature, and analyzed by CLEM. See Materials and methods for details. Representative images (fluorescence microscopy in A and B; electron microscopy in C and D; magnified electron microscopy and CLEM in E and F) are shown. White arrows indicate the mitochondrial constriction sites with Dnm1-GFP signals. Scale bars are shown in each panel.

To further investigate the mitochondrial constriction sites with Dnm1-GFP signals in *atg44*∆ cells, we performed correlative light and electron microscopy (CLEM) imaging. Based on the spatial information of Dnm1-GFP obtained by fluorescence microscopy, we analyzed the constriction sites at electron-microscopic resolution (Figure 2A–D, see Materials and methods for details). Representative images (Figure 2E,F) reveal a high electron density at the position corresponding to the Dnm1-GFP signal, which strongly suggests that the enlarged mitochondria flanking the Dnm1-labeled constriction site are interconnected by a thin mitochondrial tube. Although our CLEM imaging was not sufficient to discern the internal structure of the mitochondrial constriction sites, our results further confirmed that Dnm1 is incapable of completing mitochondrial fission in *atg44*∆ cells.

### Mitochondrial constriction sites in atg44∆ cells retain a continuous inner membrane

**Figure 3.**
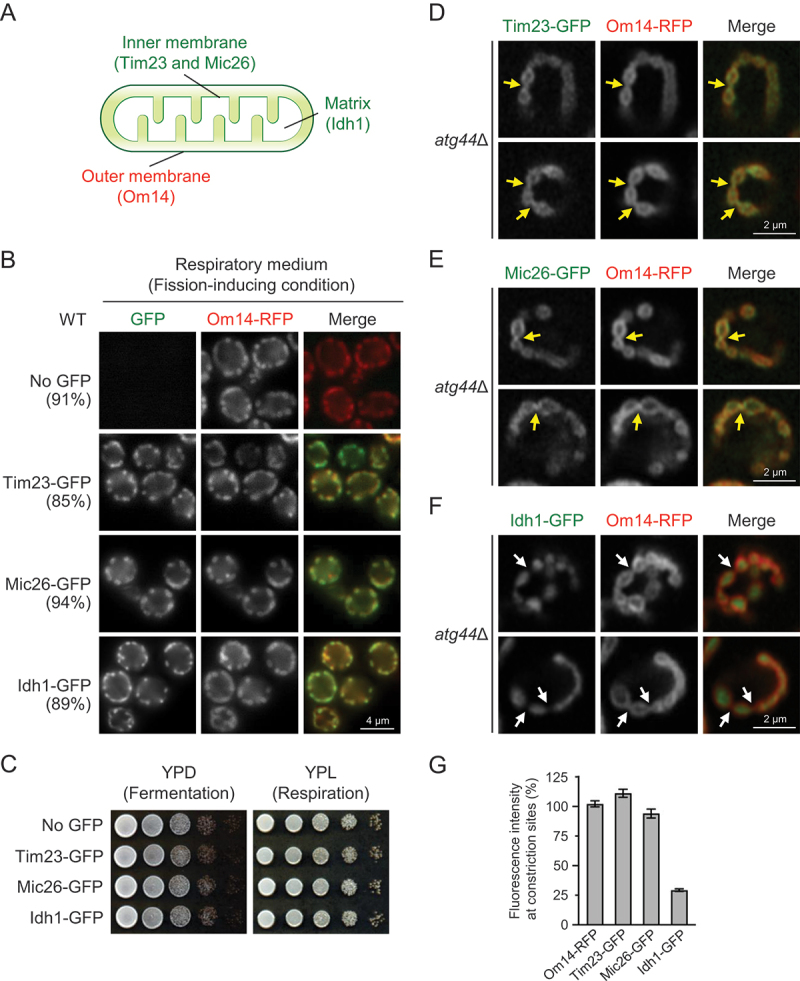
Mitochondrial constriction sites in atg44∆ cells retain a continuous inner membrane. (A) Localization of OMM, IMM, and matrix proteins analyzed in this study. (B) S. cerevisiae WT cells expressing Om14-RFP and the indicated GFP-fused proteins were cultured in YPL until mid-log phase and analyzed by fluorescence microscopy. The percentage of cells with fragmented mitochondria is shown (n = 140 cells). (C) the indicated cells were cultured in YPD until early-log phase, and their serial dilutions were spotted and cultured on YPD (1 day) or YPL (2 days) agar plates (three independent replicates). (D–F) S. cerevisiae atg44∆ cells expressing Om14-RFP and the indicated GFP-fused mitochondrial proteins were cultured in YPL until mid-log phase and analyzed by fluorescence microscopy. Yellow and white arrows indicate high and low GFP signals at mitochondrial constriction sites, respectively. (G) Bars show the fluorescence intensity (constriction sites relative to average of non-constriction sites) of the indicated RFP- or GFP-fused proteins. Om14-RFP, n = 48; Tim23-GFP, n = 53; Mic26-GFP, n = 50; Idh1-GFP, n = 49. Scale bars are shown in each panel.

To investigate whether the inner mitochondrial membrane (IMM) at mitochondrial constriction sites in *atg44*∆ cells is connected, we visualized the IMM and matrix using the established marker proteins [22]. We expressed Tim23-GFP (IMM), Mic26-GFP (IMM), or Idh1-GFP (matrix) along with Om14-RFP (OMM) (Figure 3A). We first confirmed that the fusion of GFP to these proteins did not affect mitochondrial morphology and respiratory growth in the WT background (Figure 3B,C). We then analyzed the fluorescence intensity of the GFP-fused proteins at the mitochondrial constriction sites in *atg44*∆ cells. Tim23-GFP and Mic26-GFP were well detected along with Om14-RFP at the constriction sites, while Idh1-GFP was only slightly detected at these sites (Figure 3D–G). These results suggest that the inner membrane is continuous (i.e., intact) within the constriction sites and that the constriction sites are so narrow that it becomes challenging to detect the matrix within them.

### Accumulation of Atg44 proteins is observed at the mitochondrial constriction sites

**Figure 4.**
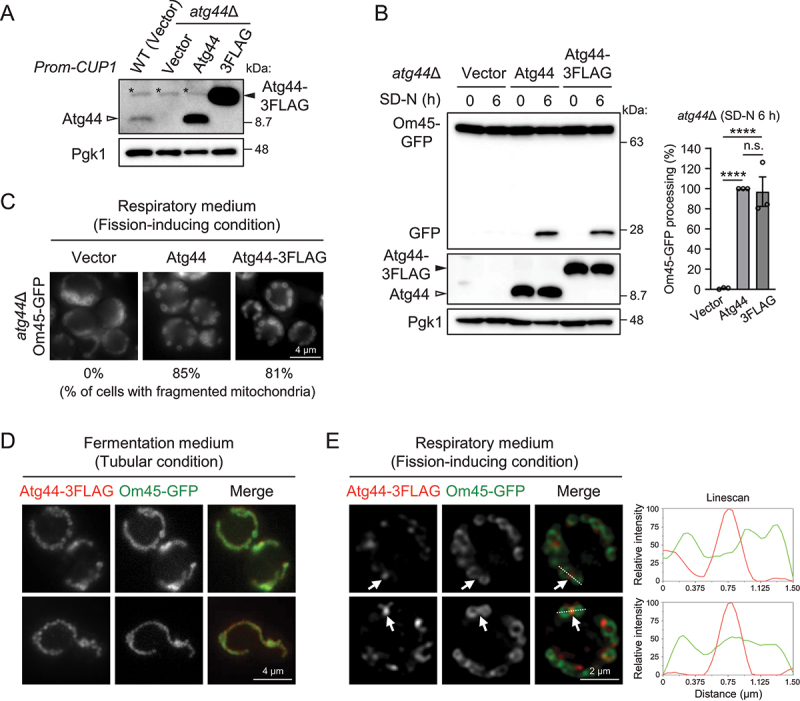
Accumulation of Atg44 proteins is observed at mitochondrial constriction sites. (A) the indicated yeast cells carrying the indicated plasmids (CUP1 promoter) were cultured in SML medium until mid-log phase and subjected to immunoblotting analysis. Endogenous Atg44 and exogenously expressed Atg44/Atg44-3FLAG were detected using anti-Atg44 antibody. Pgk1 was detected as a loading control. Representative immunoblots of three experiments are shown. Asterisks indicate non-specific bands. (B) the indicated yeast cells were cultured in SML until mid-log phase and shifted to SD-N. Cells were collected at the indicated time points, and Om45-GFP processing was monitored by immunoblotting. The value of Atg44 (6 h) was set to 100%. The results represent the mean and SE of three experiments. ****p < 0.0001, n.S. not significant (ordinary one-way ANOVA multiple comparison). (C) the indicated yeast cells were cultured in SML until mid-log phase and analyzed by fluorescence microscopy. The percentage of cells with fragmented mitochondria is shown (n = 200 cells). (D and E) atg44∆ cells expressing Om45-GFP and Atg44-3FLAG were cultured in SMD (D) or SML (E) until mid-log phase and analyzed by immunofluorescence microscopy using primary antibodies against FLAG and secondary antibodies conjugated with Alexa Fluor 594. White arrows indicate accumulated Atg44-3FLAG proteins. Dashed lines correspond to the linescans. Scale bars are shown in each panel.

Since the mitochondrial constriction sites in *atg44*∆ cells can be considered as the states just before the completion of fission, we expected accumulated Atg44 at such sites to facilitate mitochondrial fission in the case of WT cells. To verify this expectation, we analyzed the localization of functional Atg44. As our previous study did not succeed in the functional expression of fluorescence protein (GFP or RFP)-tagged Atg44, we employed an alternative method in this study. We exogenously expressed C-terminally 3FLAG-tagged Atg44 (Atg44-3FLAG) under the control of the constitutive *CUP1* promoter in *atg44*∆ cells (Figure 4A) and examined its functionality in terms of mitophagy and mitochondrial morphology. We monitored mitophagy by the vacuolar processing of a chimeric mitochondrial protein Om45-GFP to produce free GFP upon mitophagy induction [25] (Figure 4B) and analyzed the mitochondrial morphology of cells cultured in respiration medium (Figure 4C), showing that Atg44-3FLAG functions similarly to WT Atg44. Then, we performed immunofluorescence microscopy analysis and confirmed the co-localization of Atg44-3FLAG with Om45-GFP in tubular mitochondria (Figure 4D). As expected, accumulation of Atg44-3FLAG was observed at the mitochondrial constriction sites when cells were cultured in lactate medium to stimulate mitochondrial fission (Figure 4E). These results suggest that Atg44 accumulates at the site of mitochondrial fission.

## Discussion

**Figure 5.**
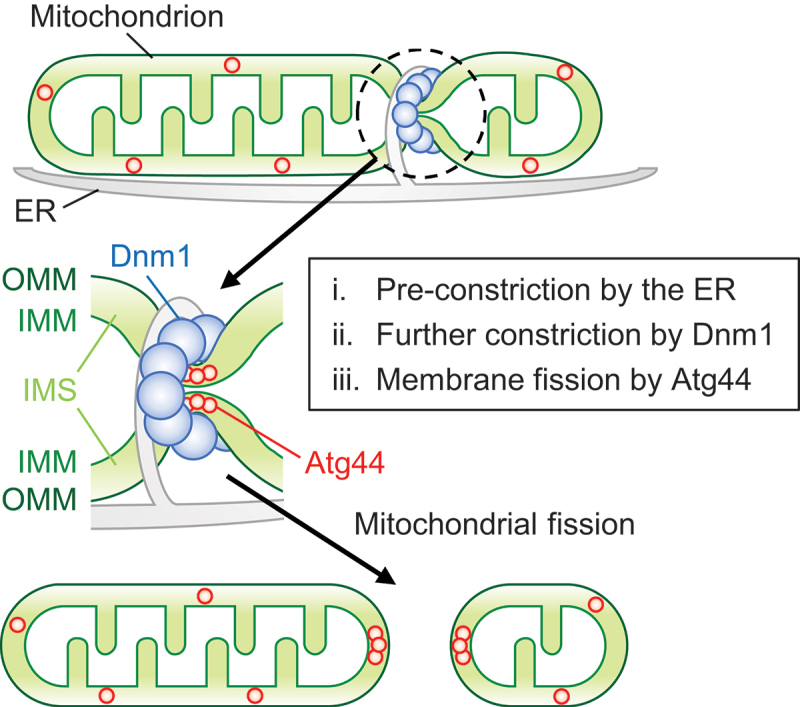
Schematic model for coordinated mitochondrial fission by Dnm1 and Atg44. See text for details.

A number of reports have demonstrated that oligomeric assembly of the dynamin-related proteins (Dnm1 in yeasts and DNM1L/Drp1 in mammals) around mitochondria causes constriction in a GTP hydrolysis-dependent manner [15,16,18–20]. However, it has remained unclear whether these fission factors eventually sever the mitochondrial membrane and whether additional mechanisms exist. In the present study, we provide evidence that recruitment of Dnm1 to the mitochondrial constriction sites is insufficient for mitochondrial fission and that the IMS protein Atg44/mitofissin is required to complete mitochondrial fission. Based on our findings and previous reports [10,11,18,23], we propose a model of mitochondrial fission (Figure 5) that involves the coordinated action of Atg44 and Dnm1: (i) The ER tubules mark fission sites and initiate pre-constriction of mitochondria; (ii) This triggers the accumulation of Dnm1, resulting in further mitochondrial constriction; (iii) In response to the Dnm1-mediated constriction, Atg44 within constriction sites binds directly to the mitochondrial membrane and facilitates membrane fission.

We observed the accumulation of Atg44 at mitochondrial constriction sites (Figure 4E). Based on this result, we speculate that Atg44 selectively associates with the mitochondrial membrane predominantly at sites of strong mitochondrial constriction, meaning that Dnm1-mediated mitochondrial constriction is the trigger for Atg44 activity. In *dnm1*∆ cells, mitochondrial fission does not occur spontaneously under homeostatic conditions even in the presence of Atg44, which supports the idea that Atg44 completes mitochondrial fission in response to Dnm1-mediated mitochondrial constriction. In mitochondrial fission during mitophagy, autophagosome closure might play a role similar to the constriction by Dnm1 [26]. Very recently, the Friedman group has reported that completion of Dnm1-mediated mitochondrial fission requires Mdi1 (Atg44) [21]. In addition, using a split-GFP approach, they have shown that GFP11_×7_-fused Mdi1, which loses its function, localizes at discrete focal structures that can be spatially linked to Dnm1-marked mitochondrial fission sites. In contrast, our Atg44-3FLAG protein is functional but overexpressed (Figure 4), leaving the possibility that it does not reflect endogenous distribution. In any case, live imaging of functional Atg44 at the endogenous level has not been reported yet. Alternative approaches are needed to understand the spatial regulation of Atg44/Mdi1 during mitochondrial fission.

In summary, our previous and present studies demonstrate that Atg44 is required not only for mitochondrial fission during mitophagy [23] but also for completion of Dnm1-mediated fission under homeostatic conditions. Although these two processes involve distinct endpoints for severed mitochondria, Atg44 plays an essential role in both processes. Therefore, the coordinated actions of factors outside the mitochondria, such as the autophagosome and Dnm1, are essential for efficient mitochondrial fission along with Atg44 inside the mitochondria. Higher organisms, including metazoans, may have functional counterparts to Atg44. The identification and functional analysis of such proteins would enhance our understanding of mitochondrial fission and provide valuable insights into human diseases derived from mitochondrial fission defects.

## Materials and methods

### Yeast strains and culture conditions

*S. cerevisiae* strains used in this study are shown in Table S1. Gene deletion and tagging were performed as described previously [27–29]. Cells were cultured at 30°C in rich medium (YPD: 1% yeast extract [Formedium, YEA03], 2% peptone [Formedium, PEP02], and 2% glucose), lactate medium (YPL: 1% yeast extract, 2% peptone, and 2% lactate), or synthetic minimal medium with glucose (SMD: 0.67% yeast nitrogen base [Formedium, CYN0402], 2% glucose, and amino acids) or lactate (SML: 0.67% yeast nitrogen base, 2% lactate [FUJIFILM Wako, 128–00056], and amino acids). YPD and YPL were used as fermentation and respiration media, respectively. Nitrogen starvation experiments were performed in synthetic minimal medium lacking nitrogen (SD-N: 0.17% yeast nitrogen base without amino acids and ammonium sulfate [Formedium, CYN0502], and 2% glucose). For growth assays, cells cultured to early-log phase in YPD medium were serially diluted and spotted onto YPD or YPL agar plates and incubated at 30°C.

### Fluorescence microscopy

*S. cerevisiae* cells expressing the indicated fluorescent proteins were cultured in YPD or YPL medium. Fluorescence images were taken using a Nikon Ti2 Eclipse microscope with a Plan Apo Lambda 100× oil objective lens and a CCD camera (MD-695, Molecular Devices) and analyzed using MetaMorph 7 software (Molecular Devices). Super-resolution images (Figure 3D–F) were taken using a Zeiss LSM880 Airyscan microscope system with a Plan-Apochromat 63×/1.4 Oil DIC and analyzed using ZEN black edition, 2.3 SP1 (Carl Zeiss). Deconvolution images are shown in Figures 1B–D, 2 A,B, and 4E. For time-lapse imaging of mitochondrial fission linked to Dnm1-GFP, yeast cells were cultured in YPD medium until early-log phase, and shifted to YPL medium in a 0.1% concanavalin A-coated 96-well glass-bottom dish (IWAKI) at 30°C using a stage-top incubator (TOKAI HIT). After 4 ± 1 h, time-lapse images were acquired at 30-s intervals for a total of 270 s. The relative fluorescence intensity of the indicated RFP- or GFP-fused proteins at mitochondrial constriction sites (Figure 3G) was calculated by dividing the intensity of constriction sites by the average intensity of non-constriction sites.

### Correlative light and electron microscopy imaging

CLEM was performed as previously described [30,31]. *S. cerevisiae atg44*∆ cells expressing Dnm1-GFP and Om14-RFP were cultured in YPL until mid-log phase and then fixed with 2% paraformaldehyde (PFA):0.1% glutaraldehyde (GA) for 30 min at room temperature. The cells were washed with 0.1 M phosphate buffer (PB, pH 7.4), placed on a glass-bottom dish (ibidi 81168), and analyzed by fluorescence microscopy. Then, cells were gently fixed with 2% GA in PB and incubated at 4°C for 2 h, followed by 0.1 M PB wash for three times, post-fixed with 1.2% potassium permanganate (KMnO_4_) in distilled water for 2 h, and washed with distilled water eight times for 1 min each. After *en bloc* staining with 1% uranyl acetate for 1 h and washed with distilled water three times, cells were dehydrated through a graded ethanol series and propylene oxide, and were embedded into 100% epoxy resin (100 g Epon was composed of 54.0 g Epon812 [Nisshin EM, 342–2], 26.5 g MNA [Nisshin EM, 3471–1], 19.5 g DDSA [Nisshin EM, 3461–1], and 1.5 g DMP-30 [Nisshin EM, 3481–1]) at 4°C for 48 h. The positions of target cells were confirmed according to the grid address on the dish and to the fluorescent images that were obtained in advance. The target cells were embedded by placing a capsule filled with pure epoxy resin upside down. After polymerization at 60°C for 48 h, the resin block was trimmed according to the address on the dish. Serial ultrathin sections (70-nm thickness) were prepared using an ultramicrotome (EM UC7, Leica Microsystems) and diamond knife (Ultra, DiATOME) were collected on formvar-coated copper single slot grids (Nisshin-EM, 2481). After stained with uranyl acetate for 10 min and with lead citrate for 5 min, sections were imaged by using TEM (Hitachi, H-7650) at an accelerating voltage of 80 kV.

### Plasmid

To construct a C-terminally 3FLAG-tagged Atg44 expression plasmid under the control of the *CUP1* promoter, a DNA fragment (*ATG44-3FLAG*) with BamHI and XhoI sites was amplified by polymerase chain reaction from yeast genomic DNA and inserted into the same sites of pCu416 [32].

### Mitophagy assay

To monitor mitophagy, the Om45-GFP processing assay was performed as previously described [25]. In brief, cells were cultured in SML medium until mid-log phase and then shifted to SD-N for 6 h. The cell lysates equivalent to OD_600_ = 0.2 units of cells were subjected to immunoblotting analysis.

### Immunoblotting analysis

Protein samples from yeast cells were resuspended in sodium dodecyl sulfate (SDS) sampling buffer (50 mM Tris-HCl, pH 6.8, 10% glycerol, 2% SDS, 5% 2-mercaptoethanol, and 0.1% bromophenol blue), incubated at 42°C for 1 h, and subjected to SDS-polyacrylamide gel electrophoresis. Proteins were transferred from polyacrylamide gels to polyvinylidene difluoride membranes (Merck Millipore, IPVH00010) using transfer buffer (25 mM Tris, pH 8.3, 192 mM glycine, 20% methanol). The membranes were blocked with phosphate-buffered saline (PBS) with Tween-20 (PBS-T; 10 mM PO_4_^3-^, pH 7.4, 140 mM NaCl, 2.7 mM KCl, and 0.05% Tween-20 [Sigma-Aldrich, P1379]) containing 5% skim milk for 1 h. The membranes were incubated with primary antibodies in PBS-T containing 2% skim milk overnight at 4°C and washed three times with PBS-T. The membranes were then incubated with secondary antibodies (Peroxidase-conjugated AffiniPure Goat Anti-Rabbit IgG [Jackson ImmunoResearch, 111-035-003] or Peroxidase-conjugated AffiniPure Goat Anti-Mouse IgG [Jackson ImmunoResearch, 115-035-003]) in PBS-T containing 2% skim milk for 1 h at room temperature and washed three times with PBS-T. Chemiluminescence signals were detected using ChemiDoc XRS+ (Bio-Rad) and analyzed using Image Lab software (Bio-Rad). Anti-GFP (Takara Bio 632380), anti-Pgk1 (Thermo Fisher Scientific 459250), and anti-Atg44 [23] antibodies were used for immunoblotting analysis.

### Immunofluorescence staining

*S. cerevisiae atg44*∆ cells expressing Om45-GFP and Atg44-3FLAG were cultured in SML until mid-log phase and were fixed with 4% PFA for 30 min at room temperature. Cells were converted to spheroplasts in spheroplast buffer (1.2 M sorbitol, 100 mM KH_2_PO_4_, pH 7.4, 100 μg/ml Zymolyase 100T [Nacalai tesque 07665–55]) for 30 min at 30°C. Spheroplasts were incubated with blocking solution containing 5% Block Ace (DS Pharma Biomedical, UKB80) and 0.05% saponin in 0.1 M PB (pH 7.4) for 30 min. Then, immunofluorescence staining was performed with primary antibodies against FLAG (Sigma-Aldrich, F1804), and secondary antibodies conjugated with Alexa Fluor 594 (Thermo Fisher Scientific, A-11020).

### Statistical analyses

Statistical analyses were performed using Prism 9 Version 9.5.1 (GraphPad Software). Welch’s t-test was used for Figures 1A–D, S1C, and S1D; ordinary one-way ANOVA multiple comparison was used for Figure 4B. *P*-values <0.05 were considered statistically significant.

## Supplementary Material

Atg44Dnm1SupplementalmaterialR4.docx
