# Supplementary material for: Atg44/Mdi1/mitofissin facilitates Dnm1-mediated mitochondrial fission
Source: Autophagy. 2024 May 31;20(10):2314–22. doi: 10.1080/15548627.2024.2360345 (PMC11423663; doi:10.1080/15548627.2024.2360345)
Supplement: Atg44Dnm1SupplementalmaterialR4.docx [file KAUP_A_2360345_SM3875.docx]

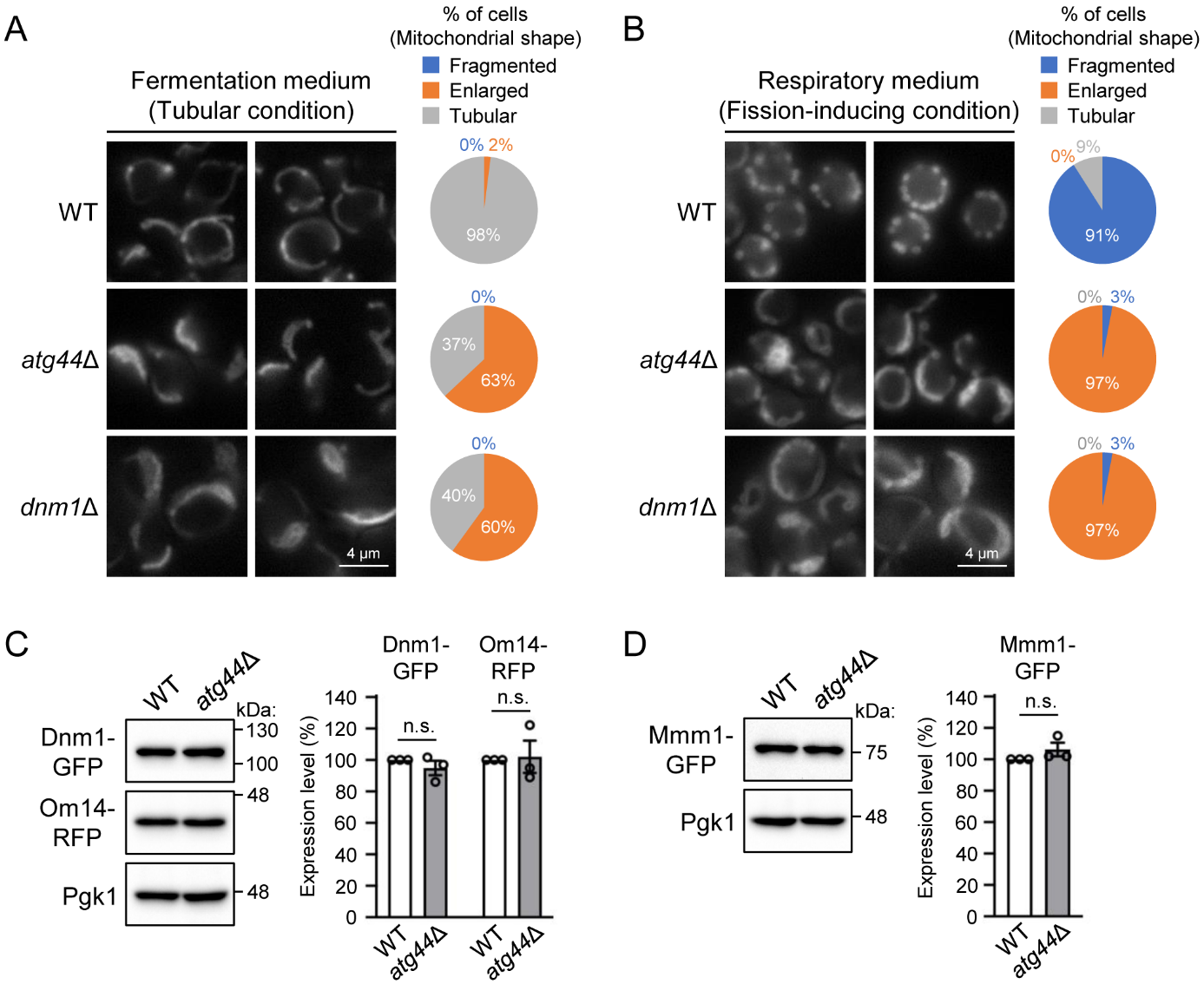


**Figure S1.** *atg44*∆ and *dnm1*∆ cells exhibit similar mitochondrial morphology and are defective in mitochondrial fission. (**A and B**) *S. cerevisiae* WT, *atg44*∆, and *dnm1*∆ cells expressing Om14-RFP were cultured in YPD until early-log phase (**A**) or in YPL until mid-log phase (**B**), and analyzed by fluorescence microscopy. Cells were classified according to their mitochondrial morphology (fragmented, enlarged, or tubular), and their ratio was quantified (n > 200 cells). Scale bars are shown in each panel. (**C and D**) *S. cerevisiae* WT and *atg44*∆ cells expressing Om14-RFP and Dnm1-GFP (**C**) or Mmm1-GFP (**D**) were cultured in YPL until mid-log phase. Expression levels (relative to Pgk1, a loading control) of Om14-RFP, Dnm1-GFP, and Mmm1-GFP were analyzed by immunoblotting. The value of WT was set to 100%. The results represent the mean and SE of three experiments. n.s. not significant (Welch’s t-test).

**
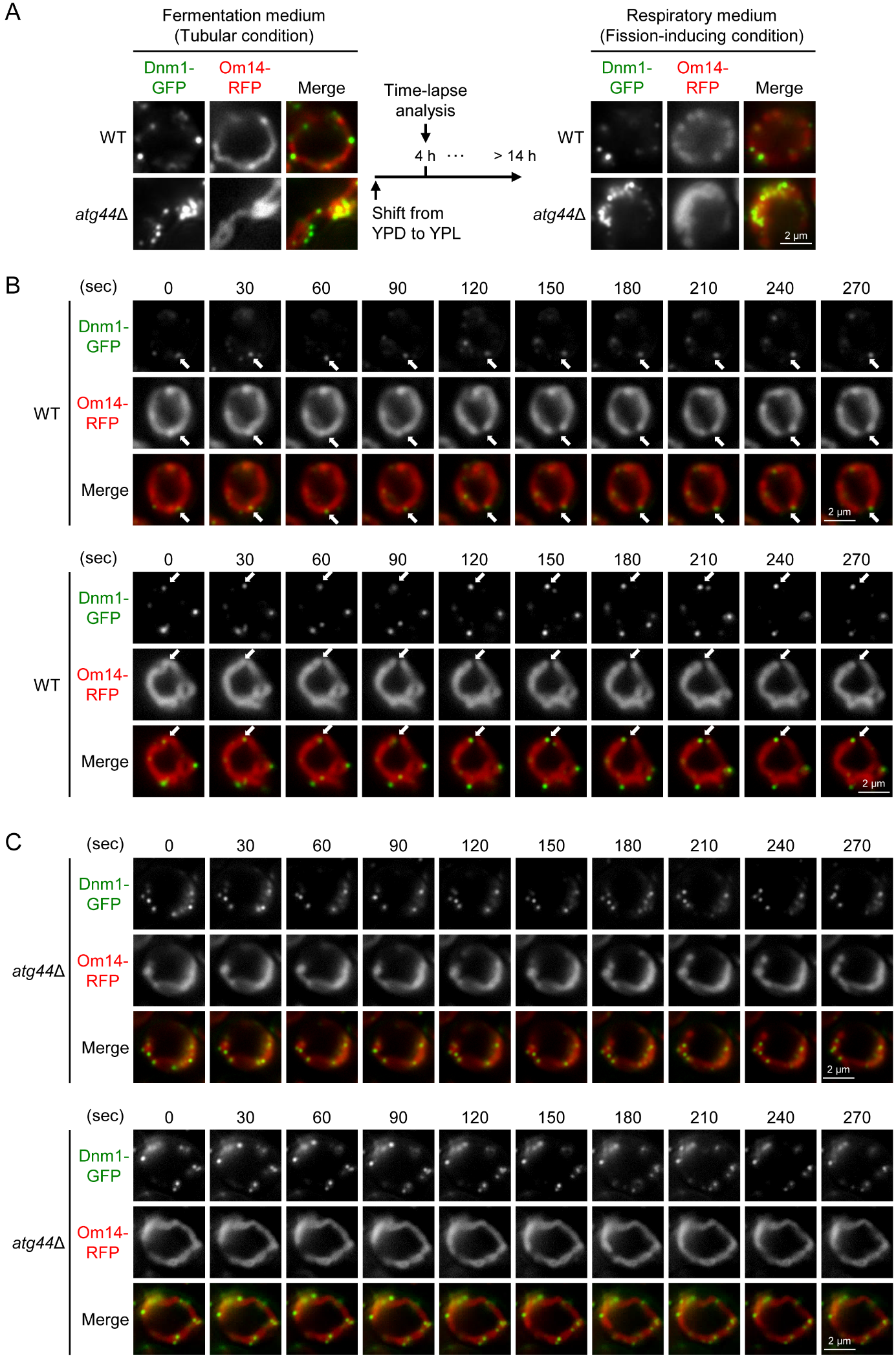
**

**Figure S2.** Additional examples for time-lapse imaging of Dnm1-mediated mitochondrial fission. (**A**) Time-lapse analyses were performed 4 ± 1 h after shifting cells from YPD to YPL medium. (**B**) WT and (**C**) *atg44*∆ cells expressing Dnm1-GFP and Om14-RFP were cultured in YPL, and fluorescence images were taken at 30-s intervals. White arrows indicate the fission sites. Scale bars are shown in each panel.

**Table S1.** *S. cerevisiae* strains used in this study.

| **Strain** | **Genotype** | **Reference** |
| --- | --- | --- |
| BY4742 | *MATα his3∆1 leu2∆0 lys2∆0 ura3∆0* | [33] |
| TKYM603 | *dnm1:: kanMX OM14-RFP::HIS3* | This study |
| TKYM607 | *OM14-RFP::HIS3* | This study |
| TKYM683 | *atg44::kanMX OM14-RFP::HIS3* | This study |
| YKF341 | *OM14-RFP::HIS3 DNM1-GFP::kanMX* | This study |
| YKF342 | *atg44::LEU2 OM14-RFP::HIS3 DNM1-GFP::kanMX* | This study |
| YKF350 | *OM14-RFP::HIS3 MIC26-GFP::kanMX* | This study |
| YKF351 | *atg44::LEU2 OM14-RFP::HIS3 MIC26-GFP::kanMX* | This study |
| YKF352 | *OM14-RFP::HIS3 TIM23-GFP::kanMX* | This study |
| YKF353 | *atg44::LEU2 OM14-RFP::HIS3 TIM23-GFP::kanMX* | This study |
| YKF354 | *OM14-RFP::HIS3 IDH1-GFP::hphNT* | This study |
| YKF355 | *atg44::kanMX OM14-RFP::HIS3 IDH1-GFP::hphNT* | This study |
| YKF383 | *atg44::kanMX OM45-GFP::HIS3* | This study |
| YKF446 | *OM14-RFP::HIS3 SEC63-GFP::kanMX* | This study |
| YKF448 | *atg44::LEU2 OM14-RFP::HIS3 SEC63-GFP::kanMX* | This study |
| YKF450 | *atg44::kanMX OM14-RFP::HIS3 MMM1-GFP::hphNT* | This study |
| YKF452 | *OM14-RFP::HIS3 MMM1-GFP::hphNT* | This study |

All strains are isogenic to BY4742.

1. Brachmann CB, Davies A, Cost GJ, et al. Designer deletion strains derived from *Saccharomyces cerevisiae* S288C: a useful set of strains and plasmids for PCR-mediated gene disruption and other applications. Yeast. 1998;14(2):115–132.

doi: 10.1002/(SICI)1097-0061(19980130)14:2<115::AID-YEA204>3.0.CO;2-2
